# Comparison of hyaluronic acid in patients with rheumatoid arthritis, systemic sclerosis and systemic lupus erythematosus

**DOI:** 10.11613/BM.2021.020701

**Published:** 2021-04-15

**Authors:** Bogdan Cylwik, Ewa Gruszewska, Ewa Gindzienska-Sieskiewicz, Otylia Kowal-Bielecka, Lech Chrostek

**Affiliations:** 1 Department of Pediatric Laboratory Diagnostics, Medical University of Bialystok, Bialystok, Poland; 2 Department of Biochemical Diagnostics, Medical University of Bialystok, Bialystok, Poland; 3 Department of Rheumatology and Internal Diseases, Medical University of Bialystok, Bialystok, Poland

**Keywords:** hyaluronic acid, rheumatoid arthritis, systemic sclerosis, systemic lupus erythematosus

## Abstract

**Introduction:**

The aim of the present study was to determine and compare the concentration of hyaluronic acid (HA) in rheumatoid arthritis (RA), systemic sclerosis (SSc) and systemic lupus erythematosus (SLE), and its correlation with parameters of disease activity and duration. The hypothesis was that HA should be increased in rheumatic diseases. We also expected that HA could be a marker of disease activity and inflammation in some of these diseases.

**Materials and methods:**

The study group comprised 149 patients with RA, SSc and SLE hospitalized in the Department of Rheumatology and Internal Diseases, Medical University of Bialystok (Bialystok, Poland) and 30 healthy controls. The concentrations of HA, C-reactive protein (CRP) and rheumatoid factor (RF) were measured using Architect ci8200; haemoglobin, platelets on Sysmex XS-800i; and erythrocyte sedimentation rate (ESR) on Sediplus S 2000 analysers. Statistical analysis was performed using Statistica 13.3 PL.

**Results:**

Hyaluronic acid was increased in RA, SLE and SSc when compared to controls (P < 0.001, P = 0.011, and P = 0.015, respectively). There were no differences in HA between rheumatic diseases (P = 0.840). Hyaluronic acid positively correlated with SLE activity (P = 0.025). In RA, HA positively correlated with ESR (P = 0.028) and CRP (P = 0.009). However, HA was not found to correlate with the duration of rheumatic diseases.

**Conclusions:**

Hyaluronic acid concentration undergoes changes in rheumatic diseases with no difference between RA, SLE and SSc. In RA, HA concentration can be a marker of inflammation, while in SLE patients an indicator of disease activity.

## Introduction

Rheumatic disorders include more than 200 different diseases from various types of arthritis to systemic connective tissue diseases. Arthritis or inflammatory arthritis, like rheumatoid arthritis (RA), is a condition which targets the synovium releasing inflammatory mediators that lead to cartilage destruction, resulting pain, stiffness, inflammation and damage to the joint (*1*). The systemic connective tissue diseases are a heterogeneous group of disorders that affect connective tissue in various organs. The most common of these conditions are systemic lupus erythematosus (SLE) and systemic sclerosis (SSc). Systemic lupus erythematosus is a typical autoimmune disease driven by autoantibodies that target multiple organ systems including joints, skin and kidneys (*1*). Another autoimmune condition is SSc that exists in two major forms: localized scleroderma (LSc) and systemic sclerosis (SSc), which may be limited or diffuse in cutaneous distribution (*1*).

Rheumatic diseases can be diagnosed based on medical history, physical examination, routine or specific laboratory tests and radiographic investigations. Results of laboratory investigations should be always interpreted in conjunction with other findings. Among routine tests, the most common are blood count with smear, indicators of acute phase, including C-reactive protein (CRP) and erythrocyte sedimentation rate (ESR), urinalysis and examination of synovial fluid (less frequently). The CRP and ESR value are often increased in systemic rheumatic diseases. It is known that a normal value does not exclude an inflammatory process, as for an increase in CRP a substantial stimulus is required. Therefore, some clinicians prefer determination of CRP concomitantly with ESR. We can say that CRP and ESR are neither diagnostic (*e.g.* CRP is only moderate higher in most connective tissue diseases, with very high concentrations in bacterial infections) nor specific markers (*e.g.* ESR increases with age); however, they are often helpful in evaluating patients with inflammatory or rheumatic diseases. It should be also remembered that joint damage entails alterations in many biochemical parameters, and therefore it may be used for diagnostic purposes. This concerns mainly the biomarkers of synovitis, which may be present in the first early stage of disease and even at pre-radiographic stages, and biologic markers of bone and cartilage metabolism that are used to diagnose and assess the progression of changes in the joints, in addition to radiographic changes that occur in the later stages of disease (*2*). Biomarkers of synovitis include N-terminal propeptide of type III collagen (PIIINP), glycoprotein 39 (YKL-40), serum cartilage oligomeric matrix protein (COMP), matrix metalloproteinases (MMPs) and their inhibitors, and hyaluronic acid (HA) (*3*).

Out of all these biomarkers hyaluronic acid was chosen due to its high concentration in synovial fluid and connective tissue, rapid serum increase during synovial inflammation caused by increased joint production and short half-life in blood (2-5 minutes) enabling easy capture of any changes (*4*). Hyaluronic acid is a high molecular weight polysaccharide that is present in various tissues and body fluids (*5*). Serum HA concentration has been found to be increased in several diseases, including some rheumatic conditions (*6*-*10*). Most of the studies have focused on the assessment of HA in RA, whereas relatively fewer reports have referred to other rheumatic diseases, such as systemic sclerosis (SSc) and SLE (*2*, *4*, *7*-*14*). We selected these three rheumatic diseases according to the type and prevalence, as one of them is a representative of inflammatory disorders and two others are connective tissue diseases.

The aim of the present study was to determine and compare the concentration of serum HA in RA, SSc and SLE, and its correlation with disease activity and duration. The hypothesis was that HA should be increased in all types of rheumatic diseases, as it occurs in high concentrations in both synovial fluid and connective tissue (*15*). During inflammation of the synovial membrane (synovitis), *e.g.* in RA, the concentrations of many compounds, like HA, increase and can reflect the activity of inflammation. Hence, we also expected that HA could be a marker of inflammation in some of these diseases.

## Materials and methods

### Subjects

This case-control study consisted of 149 patients (126 females and 23 males), aged 19-85 years (median: 54 years) with rheumatic diseases hospitalized in the Department of Rheumatology and Internal Diseases, Medical University of Bialystok, during 2018 and 2019. Patients were divided into three subgroups: RA, SSc and SLE. The diagnosis of RA was confirmed according to the American College of Rheumatology (ACR) 2010 classification criteria (*16*). According to these criteria, the diagnosis of RA is based on the presence of synovitis in at least one joint, absence of an alternative diagnosis that better explains the synovitis, and achievement of a total score of 6 or greater (of a possible 10) from the individual scores in 4 domains: number and site of the joints involved (score range 0-5), serologic abnormality (score range 0-3), elevated acute-phase response (score range 0-1), and symptom duration (2 levels; range 0-1). Rheumatoid arthritis activity was evaluated by disease activity score DAS28 (remission: < 2.6, low disease activity: 2.6 to ≤ 3.2, moderate: > 3.2 to ≤ 5.1, high: > 5.1). Patients were taking disease-modifying antirheumatic drugs (DMARDs) (methotrexate and sulfasalazine). The diagnosis of SSc was based on the ACR/European League against Rheumatism (EULAR) 2013 classification criteria (*17*). These criteria include 1 criterion that alone is sufficient for classification as SSc: skin thickening of the fingers extending proximal to the metacarpophalangeal joints. If the single criterion is not fulfilled, the point system is applied and patients with a total score of ≥ 9 are classified as having definite SSc (the maximum possible score is 19). The point system is built by adding the scores for the following clinical symptoms: skin thickening of the fingers, fingertips lesions, telangiectasia, abnormal nail fold capillaries, pulmonary arterial hypertension and/or interstitial lung disease, Reynaud’s phenomenon, and SSc-related autoantibodies. Systemic sclerosis activity was assessed by Rodnan skin score (no activity: 0, mild: 1-14, moderate: 15-29, severe: 30-39, end-stage: ≥ 40) (*18*). In turn, the recognition of SLE was made on the Systemic Lupus International Collaborating Clinics (SLICC) 2012 classification criteria (*19*). These require fulfilment of at least four criteria, with at least one clinical criterion and one immunologic criterion or lupus nephritis as the sole clinical criterion in the presence of anti-nuclear or anti-dsDNA antibodies. Disease activity was assessed by Systemic Lupus Erythematosus Disease Activity Index (SLEDAI) index at first visit to clinic (no activity: 0, mild: 1-5, moderate: 6-10, high: 11-19 and very high activity ≥ 20) (*20*). Patients were excluded from the study if they had liver diseases, hypothyroidism, chronic renal diseases, cardiomyopathy and/or other inflammatory conditions, in particular active infection and/or malignancy. Patients with more than one connective tissue disease (overlap syndromes) were also excluded. The 30 control subjects were recruited from healthy hospital workers. Detailed characteristics of patients and controls have been presented in Table 1. Written informed consent was obtained from patients after explanation of the nature of the study. The study was approved by the local research ethics committee for Medical University of Bialystok (R-I-002/392/2018).

**Table 1:** Characteristics of patients included in the study

**Demographic and** **clinical data**	**Rheumatoid arthritis** **(N = 80)**	**Systemic sclerosis** **(N = 49)**	**Systemic lupus erythematosus** **(N = 20)**	**Controls** **(N = 30)**	P*
Gender (F/M) (N)	69/11	39/10	18/2	14/16	< 0.001
Age (years)	59 (20 – 85)	52 (19 – 77)	38 (23 – 70)	25 (21 – 54)	< 0.001
Disease duration (years)	5 (0 - 35)	4 (0 – 30)	10 (1 – 21)	NA	/
DAS 28	5.76 ± 1.27	NA	NA	NA	/
Rodnan skin score	NA	8.62 ± 6.06	NA	NA	/
SLEDAI at first visit to clinic	NA	NA	15.1 ± 4.86	NA	/
RF positive (N/total)	63/80	5/49	0/20	0/30	/
Anti-CCP positive	0.84	NA	NA	NA	/
Swollen joint count	9.15 ± 3.49	NA	NA	NA	/
Tender joint count	6.88 ± 3.23	NA	NA	NA	/
VAS	66.1 ± 19.0	NA	NA	NA	/
HAQ	1.92 ± 0.76	NA	NA	NA	/
Age and disease duration are presented as median and range (min-max). Numerical data are shown as mean and standard deviation. F – female. M – male. DAS 28 – disease activity score. SLEDAI - Systemic Lupus Erythematosus Disease Activity index. RF – rheumatoid factor. Anti-CCP – anti-cyclic cytrullinated peptide antibodies. VAS – visual analog scale. HAQ – health assessment questionnaire. NA – not applicable. *Comparing controls and rheumatic patients (Chi^2^ test for gender and Mann-Whitney U test for age).					

### Materials

Blood samples (5 mL of venous blood) were collected from each patient once after a 12-hour fasting in the morning (between 07.00 and 09.00 a.m., limited physical activity) into a S-Monovette tube with a coagulation activator (Sarstedt AG & Co., Numbrecht, Germany). Radiographs were also completed at the same time on the same day. The sera for biochemical assays were separated by centrifugation at 1500xg for 10 minutes, frozen and stored at - 86 ºC until analysis. Besides serum, a portion of each blood sample was collected into the S-Sedivette tube (Sarstedt AG & Co., Numbrecht, Germany) containing anticoagulant liquid sodium citrate for determination of erythrocyte sedimentation rate and into the S-Monovette K2 EDTA tube (Sarstedt AG & Co., Numbrecht, Germany) for haematological analysis.

### Methods

Hyaluronic acid concentration (the expected value is 23 ± 17 ng/mL) was measured by the immunoturbidimetric method with WAKO reagents adapted on the Architect c8000 analyzer (Abbott Laboratories, Abbott Park, Chicago, USA). Biochemical assays such as CRP (normal values below the cut-off point of 5.0 mg/L) and rheumatoid factor (RF) (normal values below the cut-off point of 30 IU/L) were also determined by the immunoturbidimetric method on the same Architect c8000 analyzer. The concentration of anti-cyclic citrullinated peptide antibody (anti-CCP) (the results below the cut-off point of 5.0 U/mL are considered negative and ≥ 5.0 U/mL positive) was assayed by the immunochemiluminescence method on the Architect i2000 analyser (Abbott Laboratories, Abbott Park, Chicago, USA). Complete blood count (CBC) was counted on the Sysmex XS-800i analyser (Sysmex Corporation, Kobe, Japan). Normal values for haemoglobin (Hb) concentration are as follows: female: 120-160 g/L and male: 140-180 g/L, and for platelets count (PLT) 150-400 x10^9^/L. Erythrocyte sedimentation rate (normal values for women and men above 50 years are 6-11 mm/h and 3-8 mm/h) was assayed by the Westergreen method on the Sediplus S 2000 (Sarstedt AG & Co., Numbrecht, Germany). Pain intensity was measured by the visual analogue scale (VAS), ranging from 0 (very well) to 100 mm (very poor). The Health Assessment Questionnaire (HAQ) was used to assess functional ability in rheumatoid arthritis. It comprises 20 question in eight categories. A score of 0 (no difficulty), 1 (some difficulty), 2 (much difficulty or need of assistance) or 3 (unable to perform) was given to each question. The highest score in each category represents the score for that category. The sum of scores was then divided by the number of categories, yielding a total score ranging from 0 (best) to 3 (worst).

### Statistical analysis

Statistical analysis was performed using Statistica 13.3 PL (StatSoft Polska, Krakow, Poland). The results were expressed as means and standard deviations (SD) or medians and interquartile range (Q1, Q3). The Shapiro-Wilk test confirmed that HA, ESR, CRP, PLT (except in SSc), Hb (except in RA and SSc) and RF were not normally distributed. Since the majority of data were not normally distributed the differences between the study group and healthy subjects for all parameters were evaluated by non-parametric Mann-Whitney U test. To test the effect of rheumatic diseases on the concentration of HA, CRP, RF, ESR value and PLT count, the analysis of variance (ANOVA) rank Kruskal-Wallis test was performed. If P-value was statistically significant, further, the *post hoc* test for multiple comparisons was done and the P given. The Chi^2^ test was used to compare differences between genders. The correlation between variables was assessed by Spearman’s rank correlation coefficient. The results were considered to be statistically significant when P values were less than 0.05.

## Results

The results of serum HA concentrations, demographic and clinical data in healthy controls and patients with rheumatic diseases are shown in Table 1. The median of serum HA concentration was significantly higher in RA, SLE, and SSc in comparison to the control group (P < 0.001, P = 0.011, and P = 0.015, respectively). The median of ESR, CRP, and PLT concentrations in these rheumatic diseases were also significantly higher compared to the controls (except for PLT in SLE, P=0.822), whereas the median of Hb was lower in comparison to healthy subjects (Table 2). The median values of ESR and CRP in RA patients were higher compared to SSc (P < 0.001, P = 0.003, respectively). The median value of RF concentrations was significantly higher in RA compared to SLE, SSc and controls (P < 0.001 for all comparisons). In RA patients, HA concentration positively correlated with ESR (r = 0.26, P = 0.028) and with CRP value (r = 0.54, P = 0.009) but did not correlate with DAS28 (r = 0.13, P = 0.300), visual analog scale (VAS) (r = 0.22, P = 0.282), health assessment questionnaire (HAQ) (r = 0.02, P = 0.934) and the number of swollen (r = - 0.01, P = 0.966) and tender (r = - 0.09, P = 0.657) joints (Table 3, Table 4). Mean DAS28 was 5.76 ± 1.27 (high RA activity), ranging from 2.32 to 8.00 (Table 1). Among them were 82% of patients with high (mean 6.43 ± 0.74) and 18% with moderate disease activity (4.38 ± 0.51). No patients showed remission and low disease activity. There were no differences in serum HA concentrations between patients with moderate and high RA activity (P = 0.302). Further, we divided patients into 2 subgroups: with normal and elevated serum HA concentrations. The cut-off value was 55 ng/mL, which was calculated as the mean ± SD for controls. No differences in DAS28 were observed between patients with normal and elevated serum HA concentrations (P = 0.209). No differences were observed in HA concentrations between RA patients with disease duration ≤ 2 years and in patients with late-stage (46 *vs* 55 ng/mL, P = 0.904). No correlation was found between HA and disease duration (P = 0.698) (Table 4). Systemic sclerosis activity was evaluated by Rodnan skin score (mean 8.62 ± 6.06 - mild activity) (Table 1). The majority of the patients (76%) had mild disease activity (5.81 ± 3.60), while the others (24%) moderate (17.6 ± 1.67). There were no differences in serum HA concentration between patients with low and moderate SSc activity (P = 0.271). No differences in Rodnan skin score were observed between patients with normal and elevated serum HA concentration (P = 0.148). The HA concentration did not correlate with Rodnan skin score (P = 0.129) (Table 4). No differences in HA concentration were found between patients with a shorter disease duration (≤ 2 years) and those with a longer disease duration (46 *vs* 51 ng/mL, P = 0.583). No correlation was observed between HA concentration and disease duration (P = 0.774) (Table 4). Mean SLE activity assessed by SLEDAI was 15.1 ± 4.86, ranging from 8 to 26 (Table 1). There were 60% of patients with high disease activity (mean 14.6 ± 2.1), and 20% with both moderate (9.0 ± 1.15) and very high (22.5 ± 3.0) disease activity. Hyaluronic acid concentrations correlated positively with SLEDAI (P = 0.025), but did not correlate with disease duration (P = 0.403) (Table 4). Systemic lupus erythematosus patients with elevated HA had significantly higher SLEDAI index than those with normal concentrations (12.7 ± 2.36 *vs* 17.2 ± 6.32, P = 0.000). There were no significant differences in HA concentration between SLE patients with disease duration ≤ 2 years and those with late-stage (78 *vs* 43, P = 0.659). In SLE and SSc patients, HA did not correlate with indicators of inflammation (Table 3).

**Table 2:** Hyaluronic acid and laboratory parameters in healthy controls and patients with rheumatic diseases

**Group**	**Controls** **(N = 30)**	**RA** **(N = 80)**	**SLE** **(N = 20)**	**SSc** **(N = 49)**	**P**
HA (ng/mL)	35 (25 – 41)	53 (33 – 85)	44 (37 – 78)	47 (30 – 80)	< 0.001* 0.011^†^ 0.015^‡^ 0.840^§^
ESR (mm/h)	6 (5 – 7)	48 (21 – 70)	30 (20 – 45)	23 (12 – 39)	< 0.001* < 0.001^†^ < 0.001^‡^ 0.015^§^ < 0.001^‖^
CRP (mg/L)	0.7 (0.5 – 1.3)	6.4 (2.8 – 19.9)	3.9 (2.6 –13.5)	2.3 (1.0 – 6.9)	< 0.001* < 0.001^†^ < 0.001^‡^ 0.004^§^ 0.003^‖^
PLT (10^9^/L)	235 (194 – 265)	289 (214 – 348)	228 (184 – 278)	279 (235 – 328)	0.020* 0.822^†^ 0.008^‡^ 0.079^§^
Hb (g/L)	137 (133 – 142)	117 (108 – 127)	123 (104 – 131)	124 (119 – 128)	< 0.001* < 0.001^†^ < 0.001^‡^ 0.140^§^
RF (IU/L)	21 (21 – 22)	101 (33 – 258)	20 (20 – 21)	20 (20 – 20)	< 0.001* < 0.001^§^ < 0.001^‖^ < 0.001^¶^
Results are presented as median and interquartile range (Q1 - Q3). The differences between rheumatic subgroup and controls estimated by Mann-Whitney U test and the difference between rheumatic diseases by ANOVA rank Kruskal-Wallis test. For both tests, significant difference at P < 0.05. *when comparing RA and controls; ^†^when comparing SLE and controls; ^‡^when comparing SSc and controls; ^§^when comparing rheumatic diseases with each other; ^‖^when comparing RA and SSc; ^¶^when comparing RA and SLE. RA – rheumatoid arthritis. SSc – systemic sclerosis. SLE – systemic lupus erythematosus. HA – hyaluronic acid. ESR – erythrocyte sedimentation rate. CRP – C-reactive protein. PLT – platelets. Hb – haemoglobin. RF – rheumatoid factor.					

**Table 3:** Correlation coefficient between hyaluronic acid and laboratory data in rheumatic diseases

**Parameter**	**Rheumatoid arthritis**	**Systemic sclerosis**	**Systemic lupus erythematosus**
ESR	r = 0.26 P = 0.028	r = 0.12 P = 0.562	r = 0.11 P = 0.677
CRP	r = 0.54 P = 0.009	r = - 0.07 P = 0.750	r = 0.01 P=0.978
PLT	r = - 0.02 P = 0.843	r = 0.02 P = 0.927	r = - 0.05 P = 0.837
Hb	r = - 0.16 P = 0.207	r = 0.28 P = 0.168	r = - 0.42 P = 0.095
RF	r = - 0.03 P = 0.848	r = - 0.17 P = 0.478	r = - 0.29 P = 0.263
Statistical significance set at P < 0.05. ESR – erythrocyte sedimentation rate. CRP – C-reactive protein. PLT – platelets. Hb – haemoglobin. RF – rheumatoid factor.			

**Table 4:** Correlation coefficient between hyaluronic acid, inflammatory markers and clinical data in rheumatic diseases

**Clinical data**	**Inflammatory marker**	**Rheumatoid arthritis**	**Systemic sclerosis**	**Systemic lupus erythematosus**
	HA	r = 0.20 P = 0.080	r = - 0.13 P = 0.500	r = - 0.05 P = 0.839
Age (years)	CRP	r = 0.05 P = 0.826	r = 0.08 P = 0.644	r = 0.48 P = 0.043
	ESR	r = 0.29 P = 0.015	r = 0.29 P = 0.061	r = - 0.29 P = 0.237
	HA	r = 0.05 P = 0.698	r = - 0.05 P = 0.774	r = 0.22 P = 0.403
Disease duration (years)	CRP	r =-0.057 P = 0.816	r =-0.005 P = 0.974	r =-0.279 P = 0.296
	ESR	r = 0.126 P = 0.299	r = - 0.10 P = 0.507	r = - 0.17 P = 0.509
	HA	r = 0.13 P = 0.300	NA	NA
DAS28	CRP	r = 0.35 P = 0.141	NA	NA
	ESR	r = 0.80 P = 0.000	NA	NA
	HA	NA	r = 0.44 P = 0.129	NA
Rodnan skin score	CRP	NA	r = 0.18 P = 0.508	NA
	ESR	NA	r = 0.63 P = 0.004	NA
	HA	NA	NA	r = 0.53 P = 0.025
SLEDAI at first visit to clinic	CRP	NA	NA	r = 0.24 P = 0.329
	ESR	NA	NA	r = 0.58 P = 0.009
Statistical significance set at P < 0.05. DAS 28 – disease activity score. SLEDAI – Systemic Lupus Erythematosus Disease Activity index. HA – hyaluronic acid. ESR – erythrocyte sedimentation rate. CRP – C-reactive protein.				

## Discussion

To the best of our knowledge, this is a first study comparing the serum HA in three major systemic rheumatic diseases. The literature review shows that in rheumatic patients, HA was most often determined in the serum of patients with RA and there it had the highest concentration (*6*, *7*, *12*, *21*, *22*). The results of our study were comparable with those reported in literature (*7*, *12*, *21*). We found serum concentrations of HA to be higher in RA patients as compared to healthy individuals as well as SLE and SSC patients. We observed a very wide range of results, which may be caused by a broad spectrum of disease severity, number of joints involved, and also disease duration. Interestingly, half of the results were within the normal range, and some were below the lower limit. The wide range of the results in RA, and also SLE and SSC patients, might be the reason for a lack of statistically significant differences in serum HA concentration between them. Higher serum HA in RA patients may depend on increased synovial production and outflow of HA from the joints to the circulation (*23*). The excess of HA in plasma originates principally from the affected joints. Unlike other authors we did not observe the correlation between serum HA concentration and disease activity (*7*, *9*). It seems that the main reason for this was an unrepresentative study group for the disease activity (we have no patients with low RA activity and remission). Contrary to our study, other authors have seen correlation between HA concentration and the number of the joints involved in RA patients (*9*, *24*). In turn, we noticed that HA correlated with markers of inflammation, ESR and CRP. According to the results, we can say that HA is a good laboratory inflammatory marker in RA patients, but on the other hand, the strength of the correlation with ESR was weak and with CRP – moderate. It is interesting that ESR correlated with RA activity as well as with SSc and SLE activity.

Several publications about HA referring to systemic sclerosis (SSc) can be found (*8*, *25*-*28*). Systemic sclerosis is a chronic multisystem disease characterized by three pathogenic landmarks: microvascular involvement, activation of the immune system and increase in extracellular matrix deposition in the skin and internal organs (*14*). It is characterized by increased serum concentrations of different connective tissue metabolites. All authors observed higher serum HA concentration in SSc patients. Similarly, in our study the serum HA concentration was higher in comparison with controls. We did not observe differences between serum HA concentration in SSc patients and RA and SLE subjects. The serum concentrations of HA differed, ranging from normal to pathological values (range 22 - 508 ng/mL). More than half of them (52%) were within the normal range, like in RA patients. We had no subjects with severe and end-stage disease activity, which may be a limitation in our research. Hyaluronic acid in sera of SSc patients could originate from certain affected organs, joints and more specifically from the synovial fluid which is very rich in hyaluronic acid. The increased serum HA concentration may be due to abnormal stimulation by connective tissue activating substances from platelets (*28*). It was observed that the raised level of HA was more frequent in SSc patients with several clinical manifestations and immunological abnormalities, and correlated with disease severity (*8*). It is also known that HA expression in the sclerotic skin from SSc patients is more intense than that in normal skin (*8*). An important elevation of HA plasma concentrations in progressive SSc could be a serologic marker of disease severity, progression and degree of visceral involvement (*25*). We made an attempt to evaluate disease activity by Rodnan skin score. We chose this index among many clinical indicators, because SSc is characterized by skin fibrosis and visceral involvement. It is known that HA is a component of the extracellular matrix that occurs in excess in the skin and various internal organs. We found no correlation between HA and Rodnan skin score both in the whole group and in the subgroups with mild and moderate disease activity and no differences in Rodnan skin score between patients with normal and elevated HA. In turn, Yoshizaki *et al*. observed a positive correlation between HA and modified Rodnan TSS and found this index to be higher in patients with elevated hyaluronian concentrations (*8*). It seems that the reason for this discrepancy was that the majority of our patients had mild disease activity, and no individuals showed severe or end-stage activity. We can say that higher serum HA concentrations are not associated with disease activity, disorder duration and markers of inflammation in patients with SSc.

There are a few reports describing serum HA in SLE until now (*4*, *10*). The authors reported that mean serum concentration of HA in SLE was also increased, but was lower than that in RA, just like in our research. In this condition, increased HA concentrations are attributed to growth factor activity in connective tissue cells and synovial inflammation. Other authors examined HA and chondroitin sulfate in cutaneous lupus erythematosus (CLE) and found only increased HA in this condition compared to healthy controls (*10*). It is known that glycosaminoglycans, like HA, are dermal mucins and may accumulate in several inflammatory skin diseases. We have to remember that clinical symptoms in SLE include both skin and joint lesions, *i.e*. the sites that are a potential source of HA production by the connective tissue cells. Therefore, determination of serum HA concentrations may be a useful tool in the diagnosis of this condition. We showed that HA positively correlated with disease activity evaluated by SLEDAI index at first visit to clinic. This index is based on the presence of 24 weighted clinical and laboratory variables of 9 organ systems. We failed to find any publications to compare with our research. We also noted that ESR positively correlated with the activity of all three rheumatic diseases.

The limitations of our research include differences in age and sex distribution between control group and the diseased population. It is true that rheumatic diseases affect all ages and both sexes, although women are more frequently affected than men. In our study, in the three disease subgroups, the number of women was significantly higher than that of men (P < 0.001). In turn, in the control group, the number of women and men was almost the same. Similarly, the mean age of the disease-affected population was significantly higher than that in the healthy subjects (P < 0.001). Therefore, we performed further investigations to find out that there were no significant differences between serum HA concentration depending on gender (P = 0.170) and that HA did not correlate with age in the control group (P = 0.077). Another limitation of our study was the small size of the SLE and SSc groups and the unrepresentative study group for disease activity.

In conclusion, our results have demonstrated that HA concentration undergoes changes in rheumatic diseases and that no difference exists between these diseases. In RA, HA tends to correlate with age of patients and can reflect the activity of inflammation. In SLE patients, HA shows some relation to disease activity.
